# Home-Based Digital Technologies to Support Aging-in-Place for Rural African American People With Alzheimer Disease and Their Care Partners: Protocol for a Mixed Methods Feasibility Study

**DOI:** 10.2196/78623

**Published:** 2025-10-17

**Authors:** Otis Owens, Rahul Ghosal, Zachary Beattie, Jeffrey Kaye, JiaJia Zhang, Nora Mattek, Joel Steele, Thomas Riley, Nicole Sharma, Larry Frye, Leonardo Bonilha, Sue Levkoff

**Affiliations:** 1 College of Social Work University of South Carolina Columbia, SC United States; 2 Department of Epidemiology and Biostatistics Arnold School of Public Health University of South Carolina Columbia, SC United States; 3 Department of Neurology, The Oregon Center for Aging and Technology School of Medicine Oregon Health & Science University Portland, OR United States; 4 The Oregon Center for Aging and Technology School of Medicine Oregon Health & Science University Portland, OR United States; 5 School of Medicine & Health Sciences University of North Dakota Grand Forks, ND United States; 6 Life Analytics, Incorporated Woodside, CA United States; 7 Department of Neurology School of Medicine University of South Carolina Columbia, SC United States

**Keywords:** dementia, Alzheimer disease, remote patient monitoring, Black or African American, aging-in-place, digital technology, wearable electronic devices, internet of things, culturally appropriate technology, rural health, caregivers

## Abstract

**Background:**

Rural, low-income African American people have the highest Alzheimer disease and related dementia (ADRD) incidence and prevalence rates but have the least access to formal dementia care. To support individuals living with ADRD, growing evidence suggests that remote monitoring technologies can augment existing care by facilitating the completion of activities of daily living (ADLs) and maintaining communication between individuals living with ADRD and their care partners. Despite the success of remote technologies, no studies have investigated the usability, acceptability, and feasibility of these technologies among rural, lower-income African American people living with ADRD and their care partners. Understanding the potential impact of remote monitoring technology on this population can guide the development of tailored aging-in-place interventions.

**Objective:**

Among rural, low-income African American people living with ADRD and their care partners, our study, “Revolutionizing Empowerment of African Americans’ Cognitive Health Through Innovative Technology,” aims (1) to identify barriers to aging-in-place, current technology use behaviors, and attitudes toward remote monitoring technologies and (2) to examine the usability, acceptability, and feasibility of deploying a remote monitoring system in the homes of this population for supporting ADLs.

**Methods:**

In total, 10 low-income African American people living with ADRD and their care partners will be recruited from rural cities in South Carolina. Participants will complete a short web-based survey to collect demographics and their knowledge, experience, and comfort with using internet-connected devices, followed by 45- to 60-minute in-depth interviews (objective 1). In phase 2 (ie, objective 2), 10 additional pairs of participants will be recruited to use a remote monitoring system for 18 months. “Weekly Health Update” surveys will measure changes in health and time spent at home. In-depth interviews will be used at 18 months to examine the usability and acceptability of the system. Feasibility will be determined by the percentage of days data are collected across all sensors.

**Results:**

This study was approved by the institutional review board in June 2024. Recruitment for objective 1 began in January 2025. To date, 5 persons living with ADRD and their care partners have been recruited, surveyed, and interviewed about their challenges to aging with ADRD or caring for someone with the disease, technology use, and openness to remote monitoring technology. Recruitment for objective 2 is anticipated to begin in fall 2025, and data collection will conclude by May 2027. Results for objectives 1 and 2 are expected to be published in fall 2026 and winter 2027, respectively.

**Conclusions:**

Findings from the Revolutionizing Empowerment of African Americans’ Cognitive Health Through Innovative Technology study will contribute to the refinement of the Collaborative Aging Research Using Technology platform, a multisensor remote monitoring system to support the ADLs for low-income, rural-dwelling African American people living with ADRD.

**International Registered Report Identifier (IRRID):**

DERR1-10.2196/78623

## Introduction

### African American People and ADRD Prevalence

African American people are more than twice as likely to develop Alzheimer disease and related dementias (ADRD) than their White counterparts [1-3]. The proportion of African American people with ADRD is expected to increase by almost 25% over the next 4 decades [4]. Racial disparities in ADRD prevalence and incidence can be partially attributed to the higher burden of ADRD risk factors (ie, chronic diseases) and the long-term racial marginalization of African American people via structural racism. For example, African American people of any racial or ethnic group have the highest rates of hypertension, a risk factor for ADRD, than other racial groups [5]. Chronic disease occurrence and management are correlated with socioeconomic indicators such as income [6,7]. Evidence also demonstrates that rurality is associated with worse health outcomes [8,9], especially among southern, rural-dwelling African American people [10]. Therefore, African American people who reside in rural areas may be at higher risk for ADRD than those living in urban areas.

### Aging-in-Place for Rural-Dwelling Low-Income African American People With ADRD

Rural low-income African American people with ADRD are more likely to age at home. Data from the National Alzheimer’s Association show that 65% of individuals with ADRD reside in the community as opposed to living in a long-term care facility [11]. Gaugler et al [12] report that African American people are 70% more likely than White people to delay entry into a long-term care facility. These differences can be somewhat explained by factors such as African American care partners’ cultural avoidance or mistrust of medical institutions [13]. A care partner is defined as “a person who undertakes, in shared partnership with another person, an active role in the provision of health, welfare, maintenance, and protection of a person” [14]. African American people with ADRD also have a higher probability than White people with ADRD of being admitted to lower quality, mostly Medicaid-funded facilities that often lack specialized ADRD care units [15]. In rural areas, these disparities are compounded by the absence of physical access to long-term care facilities, precipitated by staffing or bed shortages, lower incomes, lack of transportation, and further distance to the nearest facility compared to urban-dwelling residents [16,17]. Thus, rural low-income African American people with ADRD could greatly benefit from interventions that can support their ability to age in place.

### Remote Monitoring for Supporting Individuals Living With ADRD

Remote monitoring can be useful for supporting aging-in-place for individuals living with ADRD. Considerable technological advances over the past 2 decades have enabled remote monitoring devices to assess an individual’s health status, movement, or other metrics and automatically transmit this information back to care providers [18]. A remote monitoring platform can be comprised of multiple wearable, mobile or wireless, or implantable technologies [18]. For persons with ADRD, these systems frequently include passive infrared or wearable sensors for detecting or monitoring activities such as mobility (eg, walking speed), wandering, sleep quality, social isolation, disease symptoms, and completion of basic activities of daily living (ADLs; eg, eating and dressing) as well as instrumental activities of daily living (IADLs; eg, taking medication and shopping) [19,20]. Conjunctively, the data from these sensors can be analyzed in real time to identify patterns indicative of cognitive decline [21] or a threat to the individual’s safety [22]. Wu et al [23] found that individuals with mild cognitive impairment (MCI) have a higher frequency of trips to the restroom and kitchen when compared to those without cognitive impairment. Similarly, findings from Alberdi et al's [24] analysis of continuous remote monitoring data show that sleep, mobility, daily routine, and outings are positively correlated with cognition and can therefore be used for tracking cognitive decline among individuals with ADRD [25,26] and also lead to a reduction in care partner distress [27,28] (identified as a key determinant of institutionalization among those with ADRD [29-31]) by enabling them to identify threats to the safety or health of the person with ADRD. Overall, these technologies may be feasible for use among individuals with early-stage ADRD and their care partners, but the routine use of remote monitoring may vary based on several factors, including the feasibility and perceived usefulness [32] of the technology and the extent to which it affects privacy [33,34].

### Study Rationale

No studies, to the best of our knowledge, have explored the feasibility of using remote monitoring among rural-dwelling low-income African American people with ADRD. A growing, but small number of studies have investigated the feasibility and acceptability of remote monitoring for urban-dwelling African American people with MCI. Specifically, 4 recent systematic reviews on the use of remote monitoring for individuals with MCI included no African American people [31,35-37]. Five other studies [38-42] included African American people, but each comprised less than 15% of African American people with total study sample sizes averaging 70. These 5 studies examined the feasibility of using remote monitoring technology for (1) assessing the validity of using walking speed as an indicator of MCI [38,39]; (2) determining if time out-of-home is a reliable assessment of a person’s cognitive, physical, and emotional state [40]; (3) discriminating between individuals with MCI and healthy individuals via a web-based questionnaire [41]; and (4) assessing the efficacy of using computer use patterns as an indicator of MCI [42]. Each study concluded that unobtrusive remote monitoring technologies could be feasibly and effectively used in homes to identify changes in cognition. More recent studies included larger samples of African American people (range 39-68), but African American people in these studies served as healthy (ie, no cognitive decline) urban comparison groups [23,43,44]. The largest of these studies was conducted to develop and test the feasibility of the remote monitoring Collaborative Aging Research Using Technology (CART) remote monitoring platform (proposed for use in this study and described in detail in the Methods section) to capture data from a variety of sensors over an extended period [44]. Their 301-participant sample included 4 diverse cohorts: rural White veterans from Oregon and Washington, low-income White people from Portland, Oregon; African American people (n=68) from Chicago; and Latinx and Hispanic residents from Miami [44]. The findings show that the CART platform was feasible for capturing scalable, real-time data from persons with and without MCI [44]. Analyses were not conducted on racial or ethnic or rural versus urban differences in feasibility. Based on these studies, remote monitoring may be feasibly used among urban-dwelling, African American people who are healthy or have cognitive impairments, but little is known about feasibility in the homes of rural-dwelling, low-income African American people. Therefore, additional research is warranted to determine if remote monitoring is appropriate for empowering this population to manage their health. The study objectives below attempt to fill this research gap. To enhance participants’ ability to remember the study name and emphasize the mission of this research, the research team created the following name and acronym: Revolutionizing Empowerment of African Americans’ Cognitive Health Through Innovative Technology, which will be referred to by its acronym “REAACH-IT” throughout the remainder of this narrative.

### REAACH-IT Study Objectives

To ascertain the readiness of rural-dwelling African American people with ADRD and their care partners to accept CART remote monitoring technologies and the challenges to implementing such technologies in rural areas, our research aims (1) to identify barriers to aging-in-place, current technology use, and attitudes toward remote monitoring technologies among low-income African American people with ADRD and their care partners and (2) to examine the usefulness, usability, acceptability, and feasibility of deploying an existing remote monitoring system in the homes of rural low-income African American people with ADRD for supporting ADLs. Related to objective 2, we will examine whether the usefulness, usability, and acceptability of the individual CART remote monitoring technologies (eg, smartwatch) are correlated with participants’ characteristics (ie, demographics), digital readiness, and feasibility. Further, we would like to determine if outcomes differ between persons with ADRD and care partners.

### Conceptual Framework

The research will be guided by the unified theory of acceptance and use of technology [45] and the dynamic tension model of aging-in-place [46]. The former suggests four key constructs: (1) performance expectancy (ie, usefulness or the degree to which a person believes that using a technology will contribute to personal gains), (2) effort expectancy (ie, usability or the degree of ease with technology use), (3) social influence (ie, the degree of importance that one perceives their social network will place on the use of the technology), and (4) facilitating conditions (ie, feasibility or the degree to which one believes that an organizational and technical infrastructure exists to support system use), along with other personal factors (eg, age and experience) contribute to an individual’s adoption of a technology for routine use [45]. The latter postulates that aging-in-place experiences are comprised of 3 interconnected constructs: identity as an older adult (ie, independence and feeling purposeful), connectedness with others (ie, socialization), and sense of place (ie, comfort or familiarity in home and community) [46]. Older adults experience threats to each of these facets of their aging-in-place experience and must negotiate a balance between them and the agency of the older adult or care partner [46]. According to Lawton and Nahemow [47], how well a person can manage their day-to-day activities (ie, adaptive functioning) depends on personal factors such as their physical and cognitive health (ie, competence) and the challenges or opportunities presented by the environment (ie, environmental press). Therefore, adaptations to the environment or the strategies used to strengthen the older adult’s ability to complete tasks could enhance the fit between the older adult and their home environment. For example, cognitive decline may compromise a person’s ability to remember to take medications (IADL), but they might negotiate this threat by adopting a pillbox with alerting capabilities. Based on these theoretical concepts, remote monitoring technologies that are feasible and perceived as useful for facilitating the completion of ADLs or IADLs may be considered acceptable for long-term use by care partners and persons with ADRD.

## Methods

### Study Reporting

This study adheres to the SPIRIT (Standard Protocol Items: Recommendations for Interventional Trials) and [48] the MMR-RHS (Mixed Methods Reporting in Rehabilitation and Health Sciences) [49] guidelines to improve the quality and transparency of our study reporting.

### Description of the CART Remote Monitoring Platform

**Figure 1:**
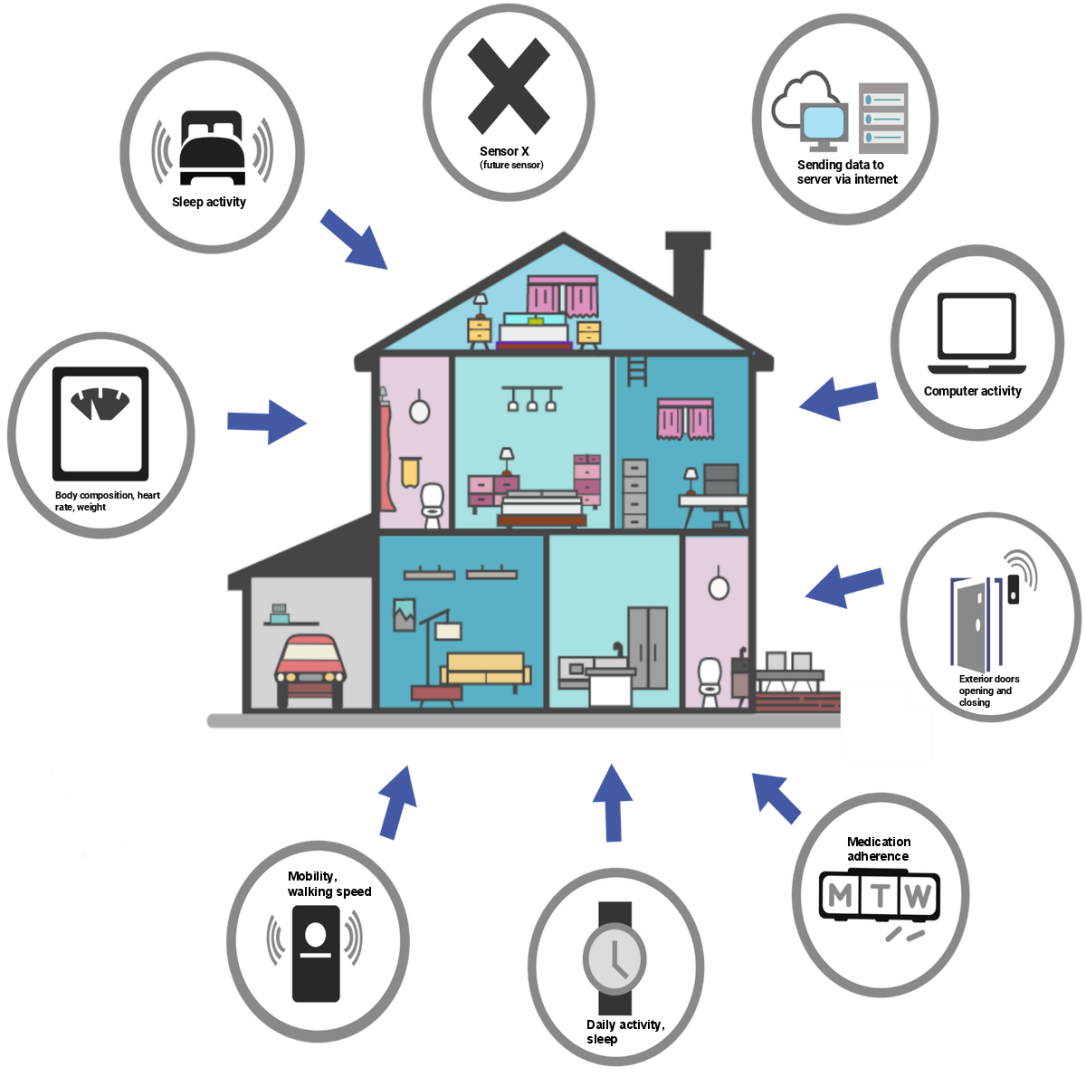
Example of a home outfitted with CART technologies. CART: Collaborative Aging Research Using Technology.

The CART platform [44,50] is a nonproprietary remote monitoring system iteratively developed since 2001 by researchers, statisticians, and software developers at the Oregon Center for Aging and Technology. Leading-edge discrete sensors, communication technologies, and data algorithms comprise the platform that enables the continuous measurement of daily activities and behaviors in the homes of older adults. It can integrate data from several commercial sensor technologies (Figure 1). Data are securely transmitted to secure servers by a hub computer using a virtual private network or via application programming interface calls to third-party vendor servers (eg, Fitbit for smartwatch data). Advanced algorithms translate the data into useful information, such as sleep and waking cycles and medication adherence. For the proposed study, the following CART technologies will be deployed and tested for feasibility: (1) smartwatch: tracks step counts and sleep metrics; (2) electronic pillbox: records timestamps when the pill compartment lids are opened and closed; (3) computer or phone activity tracker: collects the number or duration of computer sessions, phone calls, or texts and types of apps used; (4) door sensors: tracks door opening or closing; (5) motion sensors: measures walking speed and rooms visited; (6) undermattress sleep sensors: measures waking or sleeping times, sleep quality, and heart rate; and (7) scale: measures weight and body composition.

### Study Design

The study used a mixed methods, parallel convergent study design to independently gather qualitative and quantitative data, which will be merged to compare and interpret the findings in the context of our study questions.

### Setting

All research activities will be conducted in the homes of study participants in rural South Carolina.

### Participant Recruitment and Eligibility

For study objective 1, a sample of 10 pairs, that is, 10 African American people with early-stage ADRD and 10 care partners will be recruited to complete surveys and in-depth semistructured interviews. Care partners can live with or separately from the person with ADRD. However, the care partner should have an active role in supporting the person with ADRD with a strong knowledge about the challenges they are experiencing with aging in place. An additional 10 pairs of participants will be recruited for study objective 2 to test the CART platform, complete a web-based Weekly Health Update survey, and complete in-depth semistructured interviews. See Multimedia Appendices 1 and 2 for copies of the similar, but separate, interview guides for care partners and persons living with ADRD. Participants must meet the criteria outlined in Textbox 1 or will otherwise be excluded. Initial study eligibility will be determined by telephone, where participants will be asked about their race, income, and zip code. Cognition status will be determined using the 11-item Telephone Interview for Cognitive Status (total score of 40, where lower scores can denote cognitive decline) [51] and the 10-item Quick Dementia Rating System (total score of 30, where higher scores can denote cognitive decline) [52]. The Telephone Interview for Cognitive Status measures a person’s memory, orientation, attention, and language and has been validated for use in multiethnic populations [53]. Similarly, the Quick Dementia Rating System is a short, validated self-reported scale that assesses functioning in different domains of day-to-day life and has also been used in multiethnic studies [52,54-58]. Persons with ADRD who score between 23 and 33 (MCI or early-stage ADRD) [59] on the Telephone Interview for Cognitive Status and ≧1.5 on the Quick Dementia Rating System (mild or greater cognitive impairment) [54] will be eligible to participate. Care partners’ cognition will also be assessed using the Patient-Reported Outcomes Measurement Information System Short Form version 2.0—Cognitive Function Abilities Subset 8a, an 8-question survey with Likert scale responses [60]. Care partners must have a *T* score equal to or above 50 on the Patient-Reported Outcomes Measurement Information System (in addition to other criteria in Textbox 1) to be considered eligible. Any person not meeting all eligibility requirements will be excluded from the study.

**Participant inclusion criteria.**

**Person living with Alzheimer disease and related dementia (ADRD)**

- Have a self-reported memory impairment
- Telephone Interview for Cognitive Status score between 23 and 33
- Quick Dementia Rating System score ≧1.5
- Identify as African American
- Be English-speaking
- Can consent or assent to study participation
- Live in a rural area as defined by the US Census Bureau
- Have a household income below 200% of the federal poverty level
- Has a caregiver who is willing to participate in the study
- Must have home internet access (criteria for objective 2 only)
- Be comfortable with being remotely monitored for 1 month (β test) or 18 months (feasibility test; criteria for objective 2 only)

**Care partner**

- Be at least 18 years of age
- Have no self-reported memory impairment
- Patient-Reported Outcomes Measurement Information System *T* score ≧50
- Be comfortable using remote monitoring technology for 1 month (study objective 1, β test) or 18 months (study objective 2, feasibility test; criteria for objective 2 only)
- Be English-speaking
- Can legally provide consent to study participation for a person living with ADRD if they assent
- Must have internet access (criteria for objective 2 only)

Recruitment strategies used in this study will be based on the framework proposed by Dennis and Neese [61], which was designed for recruitment and retention in minority communities. The framework emphasizes community-based participatory research methods, including conceptualization, planning, and development of the recruitment plan and promotional materials in collaboration with community or institutional partners. In this ongoing study, participants will continue to be recruited in partnership with multiple local academic organizations, community-based organizations, and rural clinics. These organizations will display, distribute, mail, or email flyers to potential participants. Flyers contain contact information for the principal investigator (OO) and a QR code that links to a study interest form.

### Sample Size

The sample size for this study was selected based on project resources and the number of participants needed to conduct an exploratory study that can be used to generate hypotheses about which factors might have the greatest influence on the successful acceptance and use of the CART platform and also estimate effect sizes for a larger study in the future [62,63].

### Data Collection for REAACH-IT Study Objective 1: Formative Research

The care partner and person with ADRD will each complete separate short demographic surveys along with their questionnaires to assess “digital readiness,” a concept described by Leese et al [64] as “a participant’s knowledge, experience, and comfort level with using Internet devices.” This questionnaire contains 16 items with Likert-scale response items that range from 1=strongly disagree to 5=strongly agree. Both surveys will be administered via Qualtrics XM (Qualtrics), a web-based survey dissemination tool that can be accessed via email or administered by the principal investigator (OO). Care partners and persons with ADRD will then be engaged in separate 60-minute in-depth interviews to assess their key challenges to aging-in-place or being a care partner, current technology use behaviors, and attitudes toward remote monitoring technologies. In-depth interviews are ideal for this study because they provide a similar breadth of content as focus groups but require significantly fewer people to reach saturation [65,66].

Saturation will be determined by the point at which there is no new salient information [67]. If saturation is not reached within the 10 dyads, additional dyads will be recruited. Including care partners and persons with ADRD in separate interviews will solicit authentic individual perspectives uninfluenced by acquiescence bias or conversation domination that can occur when there is a power differential within a dyad [68]. We acknowledge that persons with ADRD may have varying challenges with word finding, abstract reasoning, concentration, and other skills necessary to follow a complex conversation [69], but there are several evidence-based strategies (eg, allowing ample time to respond, using participant wording, redirecting conversations, monitoring for signs of fatigue, making visual and sensory adaptations to interviews, and participatory techniques that prioritize the hands-on involvement of people with dementia) that can be used to effectively involve individuals with early stage ADRD in interviews [69-71]. The interview guide for this study will be based partly on a worksheet created by Tiersen et al [72] that assists care partners and those with ADRD to identify and prioritize ADL or IADL challenges that can be addressed by remote monitoring technology. The worksheet contains pictures of each daily activity (eg, taking medication) with space to indicate the level of priority for needing support for each task on a 0=not important to 10=very important scale. Based on the selected items, the interviewer will pose questions about how participants with ADRD and their care partners co-manage these tasks. Participants will also be asked about the strategies or types of technologies they use to overcome the identified challenges. To explore their openness to using remote technology, the facilitator will present pictures or physical examples of 7 remote monitoring technologies and explain how they might overcome ADL or IADL challenges. After each explanation, participants will be asked about their perceived usefulness, usability, acceptability, feasibility, and barriers to using the technology. All participants will be shown the types of data that can be derived from each of the 7 technologies, along with ways these data could potentially be visualized in a weekly, “at a glance” report. The goal is to determine the most useful data for all participants. For persons with ADRD, their comfort with having remote monitoring devices installed in their homes and the specific types of monitoring devices they deem appropriate will be ascertained. These open-ended questions will be based on both validated measures (ie, barriers to aging-in-place [72], perceived usefulness [45], usability [64], and acceptability [64]) and the expertise of the research team (ie, barriers to technology use). See Table 1 for all measures and outcomes for both study objectives.

**Table 1:** Theoretical constructs, outcomes, measures, and data collection methods.

Research objective	Theoretical constructs	Indicators or outcomes of interest	Measures	Data collection methods
1	Threats to aging-in-place^a^	Challenges with ADLs^b^ or IADLs^c^	Needs map ranking worksheet of ADLs or IADLs that can be addressed via technology Open-ended questions about challenges to ADLs or IADLs	Survey In-depth interviews
1 and 2	Performance expectancy^d^	Usefulness of technology	Open-ended questions about the usefulness of technology Perceived Usefulness Scale [45]	In-depth interviews Surveye
1 and 2	Effort expectancy^d^	Barriers to technology use	Open-ended questions about barriers to technology use Technology Usability Scale [64]	In-depth interviews Surveye
1 and 2	Social acceptance^d^	Acceptability	Open-ended questions about acceptability Technology Acceptability Scale [64]	In-depth interviews Surveye
2	Facilitating conditions^d^	Feasibility	Percentage of days of continuous data collected	Automated data tracking
1 and 2	Individual characteristics	N/A^f^	Demographics Digital readiness measure [64] Weekly Health Update	Surveys

^a^Construct from the dynamic tension model of aging-in-place.

^b^ADL: activity of daily living.

^c^IADL: instrumental activity of daily living.

^d^Construct from the unified theory on technology use and acceptance.

^e^Surveys will only be administered during feasibility testing (research objective 2).

^f^N/A: not applicable.

### Statistical Analysis for REAACH-IT Study Objective 1

Descriptive and inferential survey data statistics will be generated using SPSS (version 26.0; IBM Corp). Qualitative analysis will follow a 6-step thematic analysis approach [73] guided by our theoretical framework. The principal investigator (OO) and a coder will (1) read all transcripts for accuracy and become familiar with the data and (2) independently open-code transcripts by hand to develop a set of initial codes, which will be thoroughly discussed. The most salient codes will be used to create a codebook and to recode (ie, axially code) transcripts using NVivo (version 17.0; Lumivero). The coding team will meet to engage in an analytic process, whereby emergent themes relevant to barriers to aging-in-place for African American people with ADRD, strategies used to address these barriers, and attitudes on remote monitoring will be compared and contrasted within and between interviews. The process will include (3) discussing emergent themes, (4) reviewing and deciding on the most prominent themes, and (5) defining a final set of themes. The thematic discussion will occur only after intercoder agreement is met. If not met, the coding team will meet to discuss areas of disagreement to improve study reliability and bring in a third coder to develop consensus as needed. The final step will be (6) using the results to determine if specific CART remote monitoring technologies are appropriate for use in this population.

### Data Collection for REAACH-IT Study Objective 2: Feasibility

#### β Testing Experiment

All β testing activities will be in the homes of 2 dyads (n=4) who participated in objective 1 interviews. Persons living with ADRD must have home internet to participate in β testing, and care partners must have general internet access. β Testing will identify any unforeseen issues before full technology deployment and will take place over 1.5 months. During week 1, the principal investigator and a technology installer will deploy the 7 aforementioned CART technologies (ie, smartwatch, pillbox, computer or phone activity tracker, door sensors, motion sensors, sleep sensors, and scale). To complete the Weekly Health Update surveys and receive weekly data reports, all participants will need access to email. Participants without access to a mobile device or computer will be provided with a tablet to be used specifically for the study. The principal investigator will assist participants without a current email address in setting up an email address. Other than an explanation of the installed technologies and ensuring each participant’s access to email, no additional training is required because of the passive nature of the system. Over the next month, the research team will monitor the system to ensure that it is working properly. The care partner and person with ADRD will receive an automated weekly email with an “at a glance” report that will relay plain-language information from all sensors (eg, times medication taken each day). Persons with ADRD will complete an emailed Weekly Health Update survey (13 items) that solicits information about health events and other behaviors in the prior week, such as emergency room visits or travel [64]. The survey will be distributed via a Qualtrics survey. Qualtrics-generated reminders will be sent daily for up to 3 days before the principal investigator contacts the person with ADRD or their care partner to complete the survey. In an instance where the person with ADRD is unable to complete the survey, the care partner will be forwarded the link and asked to complete it. At the end of the month, the principal investigator will schedule separate 30-minute in-person interviews with care partners and persons with ADRD to ask about the usefulness, usability, acceptability, and barriers to using each of the installed remote monitoring technologies [64]. The principal investigator will then collect all CART technologies and compensate each participant US $100 for their participation. Qualitative data will be analyzed using a similar process to research objective 1. Saturation will not be necessary for β testing, as the goal is to assess critical barriers to implementation prior to the feasibility study. Feasibility will be calculated based on the data coverage (ie, percentage of days the technology successfully collected and transmitted data, with 80% as the measure of success). Descriptive statistics will be calculated for the Weekly Health Update survey.

#### Feasibility Study

The CART platform will be deployed as described in the β testing experiment to collect data remotely over 18 months in the homes of 10 persons living with ADRD who were not involved in objective 1 or β testing activities. Following consent, care partners and persons with ADRD will receive short surveys on a tablet computer, with assistance from the principal investigator, that solicits demographic information (10 items) and measures digital readiness (16 items) [64]. Qualtrics will send the Weekly Health Update questionnaire to persons with ADRD and reminders once per day for 3 days before the care partner and the person with ADRD are contacted by the principal investigator. Care partners and persons with ADRD will be emailed “at a glance” reports each week that provide meaningful data from all sensors (eg, quality of sleep). After 18 months, all participants will be interviewed separately about the usefulness, usability, acceptability, and barriers to using each of the remote monitoring technologies. They will also each be provided with a survey administered via a tablet that measures usefulness [45] (4 items), usability [64] (5 items), acceptability [64] (4 items), and barriers to use [64] (6 items) for each of the sensors and devices individually. This survey will be pretested with a sample of 5 volunteers prior to deployment. Feasibility will be determined by the percentage of days data are continuously collected across all remote monitoring sensors [64]. Each participant will receive US $55 per month compensation for their time and effort.

### Statistical Analysis for REAACH-IT Study Objective 2

#### Quantitative Analysis

Descriptive and inferential statistics of survey data will be generated using SPSS. To examine the relationships between dependent (usefulness, usability or barriers to use, and acceptability) and independent variables (digital readiness and demographics) across all participants, we will run Pearson correlation analyses for each of the sensors and devices. Independent 2-tailed *t* tests will be used to compare differences in independent and dependent outcomes of the care partner and the person living with ADRD. Technical feasibility success will be benchmarked to 80% of days with sensor or device activity captured and 80% with watch wear-time, pillbox lid openings, and weight taken.

#### Qualitative Analysis

Data analysis will follow a 6-step thematic analysis approach as described in objective 1. Quantitative and qualitative results will be integrated consistently with a mixed methods (convergent parallel) design to give a comprehensive picture of whether and under what circumstances remote monitoring technology is useful, usable, acceptable, and feasible among the target population. More specifically, to integrate quantitative and qualitative data, a matrix will be used to merge data by mapping survey items for usefulness, usability or barriers to use, and acceptability to qualitative data [74,75]. The matrix will enable co-coders to systematically identify areas where the qualitative and quantitative data converge and diverge. For example, if quantitative data indicate that a specific system component (eg, smartwatch) is easy to use, the team can not only investigate whether the qualitative results mirror the quantitative results but also what features of the smartwatch make it easy to use. In the instance where qualitative and quantitative data diverge across multiple participants, the team will engage in member checking to explore possible contextual explanations for these differences in quantitative and qualitative reporting. Member checking will also enhance the credibility and confirmability of the data.

### Ethical Considerations

Ethics approval was granted by the University of South Carolina’s Institutional Review Board (Study ID: Pro00137669, June 19, 2024). The study was conducted according to the principles expressed in the Declaration of Helsinki [76]. The principal investigator will first review the consent form with the care partner before requesting their signature. Once the care partner has consented and indicated their willingness for the person with ADRD to participate, persons with ADRD will undergo a brief interview to determine their capacity to give consent. The principal investigator will review a printed copy of the consent with persons who have ADRD. The person with ADRD will then be asked 5 questions from the “Evaluation to Consent” instrument (α=.81) [77], which will determine whether he or she understand factors such as “two things they will be asked to do as a part of the study” and “what to do if they no longer want to participate in the study” [77]. All answers must be correct to be deemed eligible to provide consent. If the person with ADRD can consent, they will be asked to sign a consent form after the principal investigator responds to any questions. Persons unable to consent, based on the “Evaluation to Consent” instrument, will be asked to provide assent with the care partner’s permission. Those unable to consent or assent will be excluded. The same consent process will be used for all participants (ie, study objectives 1 and 2). Rigorous measures will be implemented to ensure the confidentiality of participants’ personal information. All data will be stored on highly secure computer servers, which will be restricted to authorized study personnel. To maintain confidentiality, these encrypted data will not be directly linked to the participant’s name, phone number, or other key identifying information. Audio data will be quickly transcribed and destroyed immediately following transcription. Transcripts will also be stored on secure computer servers. Physical data storage areas will also be locked and monitored to deter unauthorized physical access. Participants who complete objective 1 will receive a one-time US $100 gift card. Participants who complete the β or feasibility study in objective 2 will receive a US $55 gift card at the end of each month for the duration of the study.

## Results

To date, we have recruited half of the participants needed for our formative study (objective 1), which is expected to be completed by September 2026. Recruitment for objective 2 is anticipated to begin in fall 2025, and data collection will conclude by May 2027. Results for objectives 1 and 2 are expected to be published in fall 2026 and winter 2027, respectively.

## Discussion

### Principal Findings

This protocol outlines a mixed methods effort to assess the feasibility of a remote monitoring platform, CART, among low-income rural-dwelling African American people living with ADRD and their care partners. We anticipate, based on our theoretical framework and prior literature, that CART technologies will be reported as useful, easy to use, and acceptable for persons living with ADRD and their care partners, but these outcomes will be highly dependent on participant’s characteristics (eg, education), digital readiness (eg, prior experience using similar technologies), and perceived performance of the technology to manage aspects of their daily lives. Study findings will contribute to a comprehensive understanding of the unique needs of low-income rural-dwelling African American people living with ADRD and their care partners. Results will elucidate under what circumstances and to what extent individual CART technologies and the platform can address the needs of persons with ADRD and their care partners. Further, findings will be valuable for the co-refinement of CART in partnership with input from community end users and stakeholders. Future dissemination of a culturally appropriate version of CART among diverse socioeconomic and racial or ethnic groups with ADRD can empower them to remain safely in their homes.

### Comparison With Prior Work

Several studies have highlighted the benefits of remote monitoring technology for persons with ADRD, such as the successful monitoring of the mobility and daily activities of persons with ADRD [31,35-37]. The CART platform, which will be tested in this study, has been feasibly and efficaciously used for continuous monitoring of multiple wellness indicators (eg, sleep, cognition, physical mobility, and social engagement) in ethnically, racially, and socioeconomically diverse cohorts in urban and rural settings [44,50]. However, the African American participants in these evaluations were cognitively sound and lived in urban areas. To our knowledge, CART nor any other comprehensive multisensor remote monitoring platforms have been evaluated among low-income, rural-dwelling African American people with ADRD. The proposed study will fill this research gap by determining the usefulness, usability, acceptability, and feasibility of deployment in the homes of rural low-income African American people with early-stage ADRD and their care partners.

### Strengths and Limitations

One key strength of this study is the use of a mixed methods study design, which will provide a methodologically rigorous means to corroborate research findings and gain a contextual understanding of factors influencing the usefulness, usability, acceptability, and feasibility of the CART platform among an understudied population. Limitations include this exploratory study’s small sample size, which limits the generalizability of study findings and also inhibits more complex statistical analyses. However, this study will still be useful in the generation of hypotheses and the estimation of effect sizes for a larger study in the future. For example, a strong positive correlation between digital readiness and usability scores, even if not statistically significant, would suggest that digital readiness is a key factor to consider in recruitment and training for a subsequent study. Likewise, observing a medium to large effect size in a small sample could indicate a practically meaningful difference between the care partner and the person living with ADRD that warrants further investigation. Guided by findings from this study, future research should evaluate the CART platform among a larger participant sample and explore the system’s capacity for providing clinically meaningful health outcomes or diagnostic data.

### Conclusions

The CART remote monitoring platform represents a promising innovation for enabling aging-in-place among rural, low-income, African American people with early-stage ADRD or their care partners. Our proposed research is critical for assessing CART’s usability, acceptability, and feasibility among a highly vulnerable population. This protocol outlines a mixed methods approach to gain a thorough understanding of participants’ lived experiences and daily needs that can be reliably supported by the CART platform. Findings will contribute to further customization of the CART platform that may lead to a greater likelihood that low-income, African American people with early-stage ADRD can safely age in place in the future. Having access to additional technological support to augment home-based and clinical care may also improve the quality of life for both persons with ADRD and their care partners.

## Appendix

Multimedia Appendix 1 Alzheimer disease and related dementia interview guide.

Multimedia Appendix 2 Care partner interview guide.

## Abbreviations

ADL: activity of daily living
ADRD: Alzheimer disease and related dementia
CART: Collaborative Aging Research Using Technology
IADL: instrumental activity of daily living
MCI: mild cognitive impairment
MMR-RHS: Mixed Methods Reporting in Rehabilitation and Health Sciences
REAACH-IT: Revolutionizing Empowerment of African Americans’ Cognitive Health Through Innovative Technology
SPIRIT: Standard Protocol Items: Recommendations for Interventional Trials
