# Supplementary material for: Home-Based Digital Technologies to Support Aging-in-Place for Rural African American People With Alzheimer Disease and Their Care Partners: Protocol for a Mixed Methods Feasibility Study
Source: JMIR Res Protoc. 2025 Oct 17;14:e78623. doi: 10.2196/78623 (PMC12579302; doi:10.2196/78623)
Supplement: Multimedia Appendix 1 [file resprot_v14i1e78623_app1.docx]

**R**evolutionizing **E**mpowerment of **A**frican **A**mericans’ **C**ognitive **H**ealth through **I**nnovative **T**echnology (REAACH-IT)

ADRD Interview Guide

Thank you for participating in this brief interview to discuss your challenges with memory loss, your current technology use behaviors, and your attitudes toward remote monitoring technologies. Our long-term goal is to use the information we learn from you and others participating in this study to further develop and test an in-home assessment system that can provide continuous activity and health monitoring for those living with memory loss or dementia using simple devices (such as watches, electronic pill boxes, and bed mats). Learning more about what you think about these devices for helping someone like yourself manage their health and reduce stress will help us decide which technologies to include in future research and what data will be most helpful for you to receive.

Do you have any questions about the purpose of the interview?

So I do not have to take any notes, we would like to gain your permission to audio record this conversation. The audio will be kept confidential and will be accessible only to people on our research team. Specifically, information gathered by this study will be stored in a locked filing cabinet and on a password-protected computer at USC. When the study findings have been analyzed and the study is complete, the audio recording will be destroyed. Only the overall results from information gathered at any point during this study will be reported for all participants collectively; no individual names will be used in any report. We will keep your name and other identifying information confidential.

Do you have any questions before we get started? (If not, start the recording and inform the participant when the recording has started).

1. How long ago did you first notice problems with your memory?
   - Probe: Can you talk more about the signs and symptoms you experienced?
   - PROMPT: Have you been to a healthcare provider, such as a doctor to discuss these memory problems? If so, talk about the conversation and the outcome. [If they have not spoken with a healthcare provider, ask Why not?]
   - Have you discussed your memory problems with family members/friends?
   - PROMPT: Do you have a family history of problems with memory?
2. How has your memory affected your ability to perform your daily activities?
   - PROMPT: [Show IADL graphic, circle each that they report having some difficulty] I am going to show you some pictures of some daily activities, and for each, tell me how memory issues have affected your (or their) ability to complete each activity.
   - PROMPT: [write down] To what extent have your memory problems affected your sleep quality, such as when you fall asleep, how long you sleep, and how rested you feel when you wake up?
   - PROMPT: To what extent have your memory problems affected your balance? Do you feel like you or fall more or are more fearful of falling?
   - PROMPT: To what extent have your memory problems affected how active you are, such as the frequency you leave the house or participate in social activities?
   - PROMPT: To what extent have your memory problems affected how often you use your phone or computer, such as sending text messages or emails?
   - PROMPT: I am going to ask you some additional questions about the activities that have been affected by your memory. [Ask about the strategies they are using to manage the difficulty with each task separately e.g., ask for help, technology use, etc.]
3. What technologies do you use on a day-to-day basis?
   - Probes: smartphone, smartpill box smartwatch, computer, smart scale? Smart watch? Security system?
   - PROMPT: Do you use any of these to help you accomplish day-to-day activities, manage your health, or remember to do something?
4. My team and I have developed an in-home assessment system to monitor that can provide continuous activity and health monitoring for those living with Alzheimer’ s Disease and Related Dementias using simple devices (such as watches, electronic pill boxes, and bed mats). These devices do not record video or audio. I would like to show you these technologies and gain your thoughts [show physical devices].
   - PROMPT: [For each technology, explain what data the sensor or technology collects then ask the following]:
     - How useful do you think this technology would be for? Why or why not?
     - Would you be willing to use this technology regularly to manage your health? Why or why not?
     - What would be some challenges to using this technology?
   - PROMPT: Which devices would be most useful? Why? Are there any devices that you would not use? Why?
5. Each of these devices provides data that can be useful for measuring health and safety.
   - PROMPT: [For each technology show a visual output of what data the sensor or technology shows then ask the following]:
     - How often would you want to see the data from each device? (e.g., only when there are big changes)
     - How would you want to receive this data (e.g., email, phone app, hard copy)?
     - How would you like the data to be displayed (graphs, numbers only) [show examples].
6. Are there technologies I have shown you that you would be uncomfortable using? Why?
7. To what extent would you want to use a system like this in the future? Why? Why Not?
8. Are there other technologies that you wish were a part of this system that we should add?
   - Probes: blood pressure monitor
   - PROMPT: Are there other conditions that you wish you could monitor using this system?
9. My long-term goal is to have this system installed in the homes of several rural African Americans, especially those with Alzheimer’s or related dementias to help with monitoring their health. What things might I need to know about this population that might help me be successful engaging them in future studies?
10. Is there anything else that I have not asked you about that you would like to tell me about your experience using the in-home assessment system?

Thank you for your time.
