# Supplementary material for: Home-Based Digital Technologies to Support Aging-in-Place for Rural African American People With Alzheimer Disease and Their Care Partners: Protocol for a Mixed Methods Feasibility Study
Source: JMIR Res Protoc. 2025 Oct 17;14:e78623. doi: 10.2196/78623 (PMC12579302; doi:10.2196/78623)
Supplement: Multimedia Appendix 2 [file resprot_v14i1e78623_app2.docx]

**R**evolutionizing **E**mpowerment of **A**frican **A**mericans’ **C**ognitive **H**ealth through **I**nnovative **T**echnology (REAACH-IT)

Care Partner Interview Guide

Thank you for participating in this brief interview to discuss your loved one’s challenges with memory loss, your current technology use behaviors, and your attitudes toward remote monitoring technologies. Our long-term goal is to use the information we learn from you and others participating in this study to further develop and test an in-home assessment system that can provide continuous activity and health monitoring for those living with memory loss or dementia using simple devices (such as watches, electronic pill boxes, and bed mats). Learning more about what you think about these devices for helping someone like your loved one manage their health and reduce stress will help us decide which technologies to include in future research and what data will be most helpful for you to receive.

Do you have any questions about the purpose of the interview?

So I do not have to take any notes, we would like to gain your permission to audio record this conversation. The audio will be kept confidential and will be accessible only to people on this research team. Specifically, information gathered by this study will be stored in a locked filing cabinet and on a password-protected computer at USC. When the study findings have been analyzed and the study is complete, the audio recording will be destroyed. Only the overall results from information gathered at any point during this study will be reported for all participants collectively; no individual names will be used in any report. We will keep your name and other identifying information confidential.

Do you have any questions before we get started? (If not, start the recording and inform the participant when the recording has started.)

1. How long ago did you notice your loved one’s problems with memory?
   - Probe: Can you talk more about the signs and symptoms they experienced?
   - PROMPT: Have they been to a healthcare provider, such as a doctor, to discuss these memory problems? If so, can you talk about the conversation and the outcome? [If they have not spoken with a healthcare provider, ask Why not?]
   - Has your loved one directly discussed their memory problems with you or other friends and family members? IF so, who?
   - PROMPT: Do you have a family history of problems with memory?
2. How has your loved ones' memory affected their ability to complete their daily activities?
   - PROMPT: [Show IADL graphic, circle each that they report having some difficulty]. I am going to show you some pictures of some daily activities, and for each, tell me how their memory issues have affected their ability to complete each activity.
   - PROMPT: [write down] To what extent have their memory problems affected their sleep quality, such as when they fall asleep, how long they sleep, and how rested they feel when they wake up?
   - PROMPT: To what extent have their memory problems affected their balance? Do you feel like they are falling more or are more fearful of falling?
   - PROMPT: To what extent have their memory problems affected how active they are, such as the frequency they leave the house or participate in social activities?
   - PROMPT: To what extent have their memory problems affected how often they use your phone or computer, such as sending text messages or emails?
   - PROMPT: I am going to ask you some additional questions about the activities that have been affected by your loved one’s memory. [Ask about the strategies they are using to manage the difficulty with each task separately e.g., ask for help, technology use, etc.]
3. **[Care Partner only].** How has your loved one’s memory loss affected your life?
   - Probe: changes in your routine schedule, stress, etc.
   - PROMPT: What are some ways that you assist your loved one?
   - PROMPT: What are some resources you have used to assist your loved one or become more knowledgeable about their condition? Have these resources been helpful? Why or why not?
4. What technologies do you use on a day-to-day basis?
   - Probes: smartphone, smartpill box, smartwatch, computer, smart scale? Smart watch? Security system?
   - PROMPT: Do you use any of these to help you accomplish day-to-day activities, manage your health, or remember to do something?
5. My team and I have developed an in-home assessment system to provide continuous activity and health monitoring for those living with Alzheimer’s Disease and Related Dementias or memory loss using simple devices (such as watches, electronic pill boxes, and bed mats). These devices do not video or audio record. I would like to show you these technologies and gain your thoughts [show devices].
   - PROMPT: For each technology, explain what data the sensor or technology collects, then ask the following:
     - How useful do you think this technology would be for you as a care partner? Why or why not?
     - Would you be willing to use this technology regularly to manage the health of your loved one? Why or why not?
     - What would be some challenges to using this technology?
   - PROMPT: Which devices would be most useful? Why? Are there any devices that you would not use? Why?
6. Each of these devices provides data that can be useful for measuring health and safety.
   - PROMPT: [For each technology, show a visual output of what data the sensor or technology shows, then ask the following]:
     - How often would you want to see the data from each device? (e.g., only when there are big changes)
     - How would you want to receive this data (e.g., email, phone app, printout/hard copy)?
     - How would you like the data to be displayed (graphs, numbers only) [show examples].
7. Are there any features of this system that you would be uncomfortable using? Why?
8. To what extent do you think that you would want to use a system like this in the future as additional support for monitoring the health of your loved one? Why? Why Not?
9. Are there other technologies that you wish were a part of this system that we should add?
   - Probes: blood pressure monitor
   - PROMPT: Are there other conditions that you wish you could monitor using this system?
10. My long-term goal is to have this system installed in the homes of several rural African Americans, especially those with Alzheimer’s or related dementias, to help with monitoring their health. What things might I need to know about this population that might help me be successful in engaging them in future studies?
11. Is there anything else that I have not asked you about that you would like to tell me about your experience using the in-home assessment system?

Thank you for your time.
